# Metagenomics Study of the Commercial Tomato Virome Focused on Virus Species of Epidemiological Interest

**DOI:** 10.3390/v17101334

**Published:** 2025-09-30

**Authors:** Zafeiro Zisi, Isabel Ruiz Movilla, Nikolas Basler, Lila Close, Lucas Ghijselings, Robby Van der Hoeven, Maria Ioanna Papadaki, Ester Rabbinowitsch, Fiona Van Reeth, Jill Swinnen, Elise Vogel, Christine Vos, Inge Hanssen, Jelle Matthijnssens

**Affiliations:** 1 Laboratory of Viral Metagenomics, KU Leuven, Department of Microbiology, Immunology and Transplantation, REGA Institute, Division of Clinical and Epidemiological Virology, 3000 Leuven, Belgium or zzi@scientia.be (Z.Z.); nikolas.basler@posteo.de (N.B.); lila.close@kuleuven.be (L.C.); mariaioanna.papadaki@kuleuven.be (M.I.P.); jill.swinnen@kuleuven.be (J.S.); 2 Scientia Terrae Research Institute VZW, B-2860 St.-Katelijne-Waver, Belgium; irm@scientia.be (I.R.M.); lgh@scientia.be (L.G.); rvdh@scientia.be (R.V.d.H.); era@scientia.be (E.R.); fvr@scientia.be (F.V.R.) or evo@dcm-info.com (E.V.); cvo@scientia.be (C.V.); 3 DCM NV, 2280 Grobbendonk, Belgium; iha@dcm-info.com

**Keywords:** virome, tomato, plant virus, metagenomics, high-throughput sequencing

## Abstract

Plant viruses have detrimental effects on commercial tomato cultivation leading to severe economic consequences. Viral metagenomics studies provide the opportunity to examine in depth the virome composition of a sample set without any pre-existing knowledge of the viral species that are present. In the present study, 101 plant samples were collected from commercial greenhouses in 13 countries in Europe, Africa, Asia, and North America between 2017 and 2024. All samples were processed with the VLP enrichment protocol NetoVIR and the obtained data were analyzed with the ViPER pipeline. Forty-three eukaryotic viral species were identified, with a median identification of 2 species per sample. The most prevalent viral species were pepino mosaic virus (PepMV), tomato brown rugose fruit virus (ToBRFV), and southern tomato virus (STV). The obtained genome sequences were used to study the diversity and phylogeny of these viruses. The three genotypes identified for PepMV showed low diversity within each genotype (96.2–99.0% nucleotide identity). Low isolate diversity was also found for ToBRFV and STV. No significant association could be found between STV identification and the presence of symptoms, questioning the pathogenic potential of STV. Three other pathogenic viral species of particular interest due to their effects on tomato cultivation or recent emergence, namely tomato torrado virus (ToTV), tomato fruit blotch virus (ToFBV), and cucumber mosaic virus (CMV), were part of the virome with low prevalence. Our study provided a comprehensive overview of the analyzed samples’ virome, as well as the possibility to inspect the genetic diversity of the identified viral genomes and to look into their potential role in symptom development.

## 1. Introduction

Plant viruses present an important threat to crop cultivation worldwide. Emerging viral diseases can result in significant reduction of yield and their estimated economic consequences can exceed 30 billion USD annually [1,2]. Current practices in the agriculture sector, such as crop cultivation of monocultures and extensive international trade, in combination with climate change facilitate the emergence and spread of known or novel plant viruses. This has led to the increasing number of viral outbreaks observed in agriculture in recent years [2,3,4,5].

With production volumes reaching 192 million tons in 2023, and a global cultivation coverage of 5.4 million hectares (ha), tomato (*Solanum lycopersicum*) is one of the world’s most economically important crops [6]. Hence, damage caused by viral infection in the form of yield loss or reduced fruit quality can have an enormous impact on the tomato value chain [7,8]. So far, 181 viral species have been associated with disease in tomato [9]. The severity of each disease depends on the infecting virus species and the climate of the growing region [1,10].

One of the viruses that constitute a well-established threat to tomato cultivation since its first detection in 1999 is the pepino mosaic virus (PepMV, *Potexvirus pepini*) [7,11]. PepMV is a member of the *Potexvirus* genus and has five genotypes, the Chilean (PepMV-CH2), the original Peruvian (PepMV-LP), the European (PepMV-EU) and the American (PepMV-US1), and the new Peruvian (PepMV-PES) [10,12]. PepMV-PES has not been identified in commercial tomatoes, it was described as infecting wild tomato species in Peru [12]. The virus currently has a worldwide distribution [11,13]. Infection with PepMV has been found to result in quality losses of 6.0–38.0% and yield losses of 5.0–10.0% in tomato crops [10,14]. Cross-protection strategies involving a mild PepMV isolate are currently being applied in many commercial greenhouses as a protective measure against natural infections with aggressive PepMV isolates [10]. Another economically important virus that emerged only in 2014 but has quickly managed to spread widely with devastating effects, is tomato brown rugose fruit virus (ToBRFV, *Tobamovirus fructirugosum*) [14]. ToBRFV, which belongs to the *Tobamovirus* genus, is able to overcome the frequently used resistance against tobamoviruses offered by the *Tm-2^2^* resistance genes in commercial tomato cultivars [15]. This devastating virus can induce losses both due to yield reduction, which can range from 15.0% to 55.0%, and due to reduced quality of the produced fruits [16]. Tomato yellow leaf curl virus (TYLCV, *Begomovirus coheni*) and tomato leaf curl New Delhi virus (ToLCNDV, *Begomovirus solanumdelhiense*) belong to the genus of *Begomovirus* and have both been associated with tomato leaf curl disease which causes significant yield reduction in tomato production [17,18]. Both viruses are mainly transmitted by *Bemisia tabaci* (whitefly) and currently have a worldwide distribution [17,18]. They can cause similar symptoms which include stunting, upward leaf curling, and reduction in leaf size [17,18]. Another vector-transmitted virus of high importance for tomato production is the tomato spotted wilt virus (TSWV, *Orthotospovirus tomatomaculae*) [19]. It is a member of the *Orthotospovirus* genus and is mainly transmitted via *Frankliniella occidentalis* (Western flower thrips) [19]. In tomato cultivation, TSWV reduces the yield, and its symptoms lower the marketability of the produced fruits [19]. Additionally, a number of other viruses have been identified in commercial greenhouses but their economic consequences in tomato cultivation are less outspoken or still being studied.

A wide variety of molecular techniques are available for fast, sensitive, and specific detection of plant viruses for routine diagnostics, such as quantitative or conventional polymerase chain reaction (qPCR, PCR), enzyme-linked immunosorbent assay (ELISA), and loop-mediated isothermal amplification (LAMP) [20]. However, these assays require prior knowledge of the viral genome sequence or serological properties and may fail in the detection of diverse strains or unknown viruses [21]. The progressive reduction in high-throughput sequencing (HTS) costs allowed the set-up of plant virus metagenomics studies, which examined the community of viruses present in a sample in its entirety, including both known and novel virus species [1,20]. Plant virus metagenomics studies generate large amounts of data that can also be used to study viral ecology and evolution, investigate outbreaks, and explore a viral population structure to identify variations that could be connected to disease etiology [20,21].

Depending on the aim of a metagenomics study, different approaches can be applied to both the wet-lab processing of the samples prior to sequencing, as well as the bioinformatics tools and pipelines used for the data analysis [22,23]. In terms of sample processing, total DNA or RNA extraction, which is frequently followed by sequence-independent rolling circle amplification or ribosomal RNA (rRNA) depletion respectively, can provide a straightforward protocol for the study of the sample’s biodiversity [22]. The downside of these approaches is that a large proportion of the obtained data are of plant and not viral origin [22]. A different approach involves the enrichment for virus-like particles (VLPs) before the isolation of nucleic acids, using filtration, centrifugation, and enzyme treatment steps to remove nucleic acids not protected by the viral coat protein [24]. This approach achieves higher ratios of viral to plant reads, although the enrichment is not limited to plant viruses, which requires a careful examination of the taxonomic classification of identified viruses [24]. Additionally, VLP enrichment protocols add to the preparation complexity and do not allow the study of non-encapsidated viruses or viroids [22]. Double-stranded RNA (dsRNA) isolation can also enrich for viruses as these molecules are not naturally occurring in plants, while small interfering RNA (siRNA) isolation allows an in-depth study of viruses that trigger the RNA interference (RNAi) pathway in plants [22]. Both dsRNA and siRNA approaches are laborious and negatively select for many viruses such as single-stranded and DNA viruses, in case of dsRNA isolation, and viruses that produce silencing suppressors, in case of siRNA isolation [22,24]. In terms of bioinformatics analysis, although there aren’t any methodological standards established currently, all pipelines include an initial quality control step of the raw data, followed by trimming the high-quality reads, removing the sequencing adapters, and filtering the processed reads [23]. The viral genomes are then obtained either by mapping the reads to an existing database or by performing *de novo* assembly [23]. Finally, the assembled genomes are taxonomically classified by comparison to reference databases [23].

Considering the high impact plant viruses can have on commercial tomato production worldwide, the aim of the present study was to analyze the virome of commercial tomato production in protected cultivation with a focus on viruses of epidemiological interest. In order to perform a comprehensive phylogenetic analysis of epidemiologically interesting viruses, such as PepMV and ToBRFV, positive detection cases were preferentially included in the study. Hence the study was not performed on a completely random sample set. One hundred one samples were collected from commercial greenhouses in 13 countries in Europe, Africa, Asia, and North America, from 2017 until 2024, from both symptomatic and asymptomatic plants. All samples were processed using the Novel Enrichment Technique of VIRomes (NetoVIR), a VLP enrichment protocol [25], and the generated data were analyzed using the in-house developed bioinformatics pipeline Virome Paired-End Reads (ViPER) [26]. The virome study includes an assessment of the viral abundance and diversity, the study of the phylogeny of the identified viruses, and an evaluation of the possible association between virus identification and disease etiology.

## 2. Materials and Methods

### 2.1. Sample Selection

The sample collection contained 101 plant material samples, more specifically 83 leaf, 13 fruit, and 5 sepal samples, collected for diagnostic purposes from commercial tomato greenhouses in Azerbaijan, Belgium, Czech Republic, France, Germany, Greece, Italy, Morocco, the Netherlands, Slovakia, Switzerland, the United Kingdom, and the United States of America. Sampling was performed between 2017 and 2024 from symptomatic or asymptomatic tomato (*Solanum lycopersicum*) plants. The samples were collected at different developmental stages during crop monitoring upon observation of symptoms indicative of viral infection either by growers or by crop advisors. All samples were stored at −20 °C after collection. Along with the samples, metadata were collected regarding the sampling time point, country of collection, and presence of symptoms.

The sample selection aimed to: (i) maximize the number of countries of collection, (ii) maximize the collection timeframe, (iii) maximize samples with epidemiologically interesting conclusive diagnostics results, especially cases of PepMV and ToBRFV infection, to study their phylogeny, (iv) include samples with inconclusive diagnostics, (v) for each symptomatic sample include, when available, an asymptomatic sample from the same greenhouse as a ‘healthy’ control. The selection rationale resulted in a sample set with a strong bias towards PepMV and ToBRFV cases.

**Figure 1:**
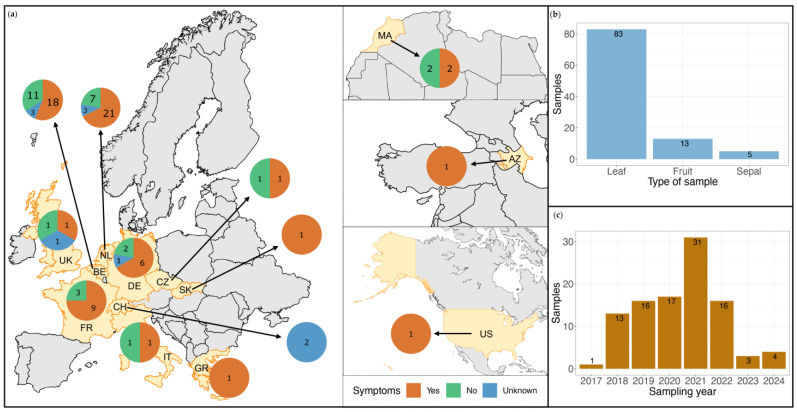
Sample-set overview. (**a**) The 13 countries where the analyzed samples were collected are indicated in yellow on the map and labeled with their two-letter code: BE—Belgium, NL—the Netherlands, DE—Germany, UK—the United Kingdom, CH—Switzerland, FR—France, MA—Morocco, IT—Italy, US—the United States of America, CZ—Czech Republic, SK—Slovakia, GR—Greece, AZ—Azerbaijan. The number of collected samples and their symptom status are presented in the pie charts of each country. The number of samples is indicated in black numbers and the symptom status is indicated in color: orange for symptomatic samples, green for non-symptomatic samples, and blue for samples for which the presence of symptoms was unclear; (**b**) Sample type overview of the sample-set; (**c**) Sampling year overview of the sample-set.

An overview of the 101 plant samples that were selected for this study is presented in Figure 1. Each sample was analyzed separately. Supplementary Table S1 presents the complete sample list and metadata collected for the sample set. The sample pairs collected from the same commercial greenhouses have been assigned numbers to enable comparisons.

### 2.2. Molecular Viral Detection

The molecular detection of viral species of epidemiological interest was performed in the framework of diagnostics analysis upon request of a grower or crop advisor when symptoms could be linked to a viral infection and in the framework of confirming the HTS results. These assays were performed on the original plant material.

For the analysis of plant material samples collected until 2022, 100 mg were collected from the original sample and homogenized in 600 μL of Buffer RTL Plus with 1.0% β-mercaptoethanol (RNeasy Plus kit, Qiagen, MD, USA) in Bead Ruptor 24 (Omni International, Inc., Kennesaw, GA, USA), for two cycles of 20 s at 5.5 m/s with a dwelling step of 30 s. The homogenization step was followed by RNA extraction using the RNeasy Plus kit (Qiagen, MD, USA).

For the analysis of plant material collected from 2023, 100 mg were collected from the original sample and diluted in 800 μL of MagMAX Plant RNA kit (ThermoFisher Scientific, Frederick, MD, USA) lysis buffer with DTT and homogenized with 1.4 mm ceramic beads (Qiagen, MD, USA) in Bead Ruptor 24 (Omni International, Inc.), for two cycles of 20 s at 5.5 m/s with a dwelling step of 30 s. The samples were then extracted using the MagMAX Plant RNA kit (ThermoFisher).

Viral detection was performed using reverse transcription-quantitative polymerase chain reaction (RT-qPCR) protocols with specific primers for each virus (Supplementary Table S2) [27,28,29,30,31,32,33]. A positive detection for the PepMV-EU/PepMV-LP assay was followed by differentiation between the two genotypes using a Restriction Fragment Length Polymorphism (RFLP) protocol described by Hanssen et al. [34] when the viral titer was within the RFLP detection range.

### 2.3. Sample Processing and HTS

The HTS processing was performed using the NetoVIR protocol as it can purify VLPs from complex samples and can easily be adapted to different sample types and large-scale studies [25,35,36]. The NetoVIR protocol was used with an adapted sample homogenization approach. In short, for each plant material sample 100 mg was collected, diluted 10.0% with PBS, and homogenized with 2.8 mm ceramic beads in Precellys Evolution Touch homogenizer, for two cycles of 10 s at 5000 rpm with a dwelling step of 5 s. The homogenate was used as the starting material for the VLP enrichment step of the NetoVIR protocol.

The VLP enrichment was performed by centrifuging the samples for 3 min at 17,000× *g*, followed by filtration of the supernatant through a 0.8 µm PES filter. The flow-through was treated with two nuclease enzymes, Benzonase nuclease (Millipore) and Micrococcal nuclease (New England Biolabs), to digest any nucleic acids not protected by the viral coat protein.

Following the VLP purification step, 140 μL were collected for each sample and nucleic acids were extracted using the QIAamp Viral RNA mini kit (Qiagen, MD, USA). Reverse transcription and random amplification were then performed on the extracted molecules using the Whole Transcriptome Amplification kit (WTA2, Sigma Aldrich), followed by purification of the amplification products. The sequencing libraries were prepared with the NexteraXT Library Preparation Kit (Illumina, San Diego, CA, USA) with unique double barcodes and cleaned up with a 1.8 ratio of Agencourt AMPure XP beads (Beckman Coulter, Inc., Brea, CA, USA). Sequencing was performed on the NovaSeq6000 platform (Illumina) and on the Element Bio AVITI instrument for 300 cycles (2 × 150 bp paired ends), resulting in an average of twenty-two million reads per sample.

### 2.4. Bioinformatics Analysis

The HTS data were analyzed using the in-house developed bioinformatics pipeline ViPER [26] (Supplementary Table S3 presents analysis details). The obtained paired-end reads were trimmed using Trimmomatic v0.39 [37] to remove the low-quality bases of each read as well as the sequencing adapters. The quality trimming resulted in an average of 18.2 million remaining reads per sample.

Then the reads were *de novo* assembled into long contiguous sequences (contigs) with a minimum length of 500 bp using metaSPAdes v4.0.0 [38] with k-mer sizes of 21, 33, 55, 77. The assembly for each sample was performed in triplicate: (i) on the full dataset, (ii) on 10.0% of the reads, and (iii) on 1.0% of reads. The triple assembly step was implemented to reduce the possibility of viral genome fragmentation during assembly due to very high coverage. The contigs produced by the three assemblies were then clustered together to remove redundancy. Clustering was performed using a combination of BLAST+ v2.14.0 [39] and two clustering scripts (anicalc.py and aniclust.py) from the CheckV v1.0.1 [40] package. The resulting contigs set per sample was used in a second clustering across the complete study to obtain a non-redundant contig collection. The second clustering step was also performed using the above-mentioned clustering scripts from the CheckV v1.0.1 [40] package with average nucleotide identity (ANI) for clustering contigs at 95.0% and lowest coverage at 85.0%. The longest contig in each cluster is deemed the cluster representative.

The non-redundant contig collection was used to identify and classify eukaryotic viruses using DIAMOND v2.1.8.162 [41] with the “—sensitive” setting against the NCBI NR protein sequence database, and KronaTools v2.8.1 [42] using the lowest common ancestor approach. Viral abundances per sample were determined by mapping the quality trimmed reads to the non-redundant contig collection with bwa-mem2 v2.2.1 [43]. For each sample, the coverage percentage of the non-redundant contig collection, as well as the consensus sequence for each classified contig were determined using SAMtools v1.17 [44].

For reporting, two thresholds were set for the identification of eukaryotic viral findings that were classified up to the species level. The first threshold was a 70.0% coverage of the cluster representative contig of the non-redundant contig collection for all assembled contigs, and the second was a viral read relative abundance of 0.005% across each sample. The threshold percentage was set to ensure that low abundance contaminating reads would not be included in our reporting, while sensitive viral detection would still be possible across the different sample types. The selection was based on the positive RT-qPCR results of the confirmatory assays. All viruses with lower contig coverage and/or read relative abundance were considered absent.

**Figure 2:**
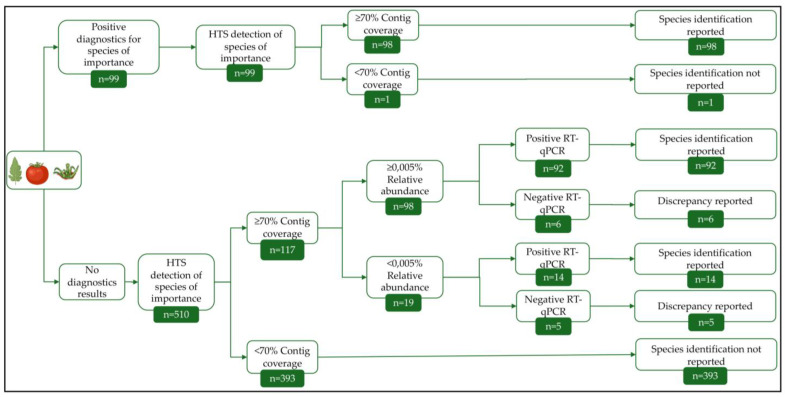
Modified identification reporting decision tree for the 6 viral species of interest for tomato cultivation (PepMV, ToBRFV, STV, ToTV, ToFBV, CMV); n indicates the number of cases fulfilling the decision criteria for each step.

Six of the viral species identified in this study were of particular interest either due to their putative devastating effects in tomato cultivation, because their role in disease etiology is understudied, or because of their recent emergence. The 6 species of interest were: PepMV, ToBRFV, southern tomato virus (STV, *Amalgavirus lycopersici*), tomato torrado virus (ToTV, *Torradovirus lycopersici*), tomato fruit blotch virus (ToFBV, *Bluenervirus solani*,), and cucumber mosaic virus (CMV, *Cucumovirus CMV*). The identification reporting process was modified to increase the identification sensitivity for these viruses. The modified process along with the number of cases fulfilling the criteria for each step are presented in the decision tree in Figure 2. In cases where positive diagnostics results (RT-qPCR or RT-PCR) were available prior to HTS for the 6 species of interest, identification was reported based on the 70.0% coverage of the cluster representative contig. For viruses with multiple segments, the high-quality trimmed reads were mapped with bwa-mem v0.7.17 (r1198) [43] against each segment of the reference viral genomes. For ToTV, mapping was done against NC_009013.1 and NC_009032.1, for ToFBV against NC_078392.1, NC_078395.1, NC_078394.1, and NC_078393.1, CMV against NC_002034.1, NC_002035.1, and NC_001440.1. For newly identified cases (no prior RT-qPCR or RT-PCR results), we performed RT-qPCR on the sample to confirm the viral identification. The assays were performed as described in 2.2 on the original material for plant samples. This process was followed for contigs both above and below the 0.005% viral read relative abundance. Identification cases that were confirmed with RT-qPCR were reported. Non-confirmed cases were reported as discrepancies in the analysis. The nearly complete genome sequences for the 6 viruses of interest were collected from all the samples for submission in the NCBI GenBank database. The web Blastn suite was used to confirm the differentiation between the three PepMV genotypes identified.

For the pairs of symptomatic and non-symptomatic samples collected from the same commercial greenhouse with STV identification in at least one member of the pair, statistical significance analysis was conducted on the obtained reads differences between symptom statuses using Wilcoxon signed-rank test for paired samples using function wilcox.test() in R v4.1.2.

The HTS analysis results were visualized using an in-house R script, Biorender.com, Microsoft PowerPoint, and Adobe Photoshop.

### 2.5. Phylogenetic Analysis

The assembled viral genome sequences for monopartite viruses along with the consensus viral segment sequences obtained via mapping for the multipartite viruses obtained in this analysis were used to create sequence collections for each of the identified viral species of interest for tomato cultivation. The nearly complete genome sequence collections of PepMV, ToBRFV, and STV were combined with publicly available complete genome sequences in the NCBI GenBank database (visited on 23 December 2024, Supplementary Table S4). The sequences of each collection were aligned using the MAFFT software [45], and maximum-likelihood phylogenetic trees were constructed using the IQ-TREE v1.6.12 software [46] using 1000 bootstrap replicates. ModelFinder [47] (-m TEST option) was used to select the appropriate substitution model. The PepMV phylogenetic tree was constructed using the GTR + F + I + G4 substitution model, the ToBRFV phylogenetic tree using TVM + F + I + G4, and the STV phylogenetic tree using HKY + F + I. The phylogenetic trees were visualized using the FigTree v1.4.4 software (http://tree.bio.ed.ac.uk/software/figtree/, accessed on 25 November 2018). The nearly complete consensus sequences for the 4 segments of ToFBV obtained from the 4 samples were aligned with publicly available sequences of the virus available in the NCBI GenBank database (visited on 7 April 2025, Supplementary Table S4) and nucleotide sequence identity was evaluated using web Blastn suite.

## 3. Results

### 3.1. Commercial Tomato Virome Overview

**Figure 3:**
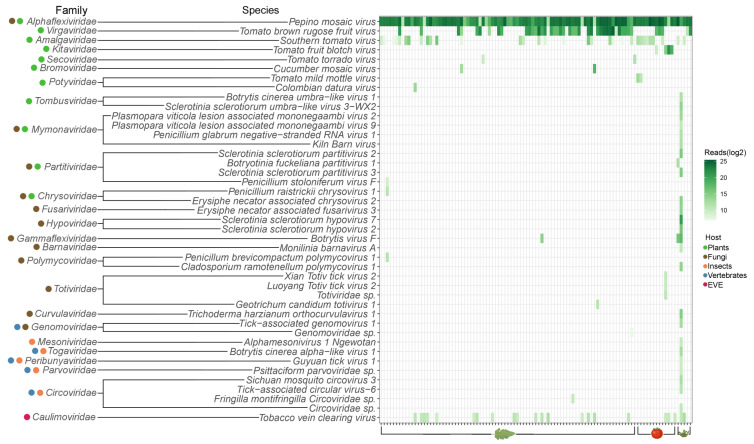
Virome composition overview. *x*-axis: Analyzed samples by sample type order: leaf (83), fruit (13), sepal (5); *y*-axis: Identified viral species and family and their known host(s) for each viral family as determined via ICTV and ViralZone [48] and indicated with colored circles next to each family name. The heat map presents the eukaryotic viral species identification for each sample. For 6 viral species of interest for tomato cultivation (pepino mosaic virus, tomato brown rugose fruit virus, southern tomato virus, tomato fruit blotch virus, tomato torrado virus, cucumber mosaic virus identification was reported for findings with ≥70.0% cluster representative contig coverage and positive RT-qPCR results. For all other eukaryotic viral species, identification was reported for findings with a reads relative abundance of ≥0.005% that covered ≥70.0% of the cluster representative contig. The color gradient boxes indicate the reads obtained for each viral species for each sample on a logarithmic scale, with darker shades corresponding to a higher read count and lighter shades to a lower count. The red arrow indicates the sample diverging from the median identification of 2 viral species per sample.

For 68 of the 101 analyzed plant samples (collected from commercial tomato greenhouses), viral reads represented the majority of the obtained reads (≥50.0%). The analysis of all 101 samples resulted in the identification of 43 viral species belonging to 25 families. The findings for each sample are presented in Figure 3. The websites of the International Committee on Taxonomy of Viruses (ICTV) and ViralZone [48] were used to determine the known host range of viruses belonging to these 25 viral families identified in the HTS analysis (Figure 3). Plants are known to be the only hosts for 7 families, fungi for 7, and insects for 1 (Figure 3). Some viral families are known to contain members able to infect more than 1 host group. Four such families were found containing members infecting both plants and fungi, 1 infecting both fungi and vertebrates, and 4 infecting both insects and vertebrates (Figure 3). Additionally, the *Caulimoviridae* family of plant viruses was identified to correspond to endogenous viral elements (EVEs) integrated into the tomato genome (Figure 3).

The median of viral species identified per sample was 2, with only 1 sample diverging: S16 (indicated with a red arrow in Figure 3). Twenty-six species belonging to 18 families were identified in S16, a non-symptomatic sepal sample. Ten of those families had fungi as hosts indicating a potential fungal infection in the sampled plant.

**Figure 4:**
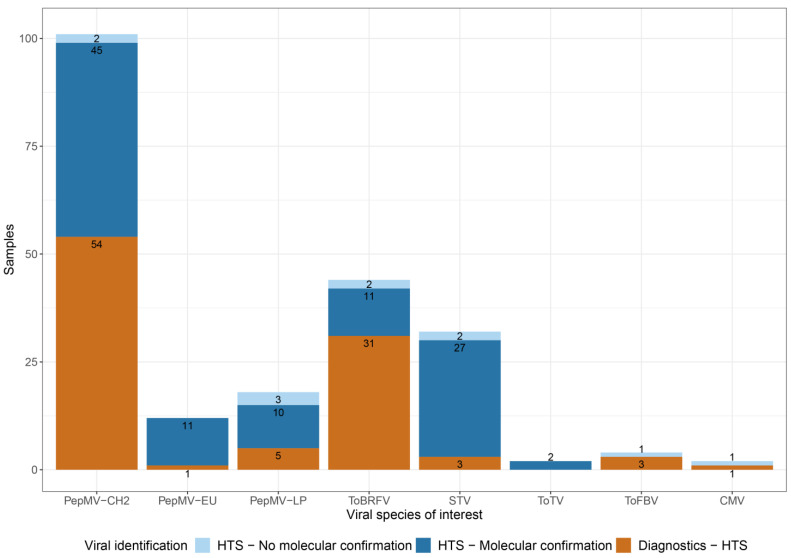
Identification overview of 6 viral species of interest for tomato cultivation. *x*-axis: Viral species: pepino mosaic virus Chilean genotype (PepMV-CH2), pepino mosaic virus European genotype (PepMV-EU), pepino mosaic virus Peruvian genotype (PepMV-LP), tomato brown rugose fruit virus (ToBRFV), southern tomato virus (STV), tomato torrado virus (ToTV), tomato fruit blotch virus (ToFBV), cucumber mosaic virus (CMV); *y*-axis: number of samples. The bars correspond to cases of viral identification in the sample set for each of the 6 viral species of interest. The bars are colored to distinguish between three identification types: cases of initial molecular detection during diagnostics analysis that were confirmed by HTS are colored orange, cases of initial detection during the HTS analysis that was confirmed afterward by RT-qPCR are colored dark blue, cases of initial detection during the HTS analysis that could not be confirmed with RT-qPCR are colored light blue.

The obtained virome contained 6 viral species of interest for tomato cultivation: PepMV, ToBRFV, STV, ToTV, ToFBV, and CMV. These viruses are either of epidemiological interest for tomato cultivation or have recently emerged. Additionally, the role of some of those species in symptom development is not well-characterized. The confirmation process for the HTS findings that are reported in this study for these species is described in Figure 2. Findings with positive molecular diagnostics results generated prior to HTS were directly reported. Findings for which diagnostics were not requested, or results were inconclusive, were confirmed by performing RT-qPCR after HTS on the original plant sample material (Figure 4, Supplementary Table S1).

PepMV and ToBRFV were the most prevalently identified viruses (Figure 3). The PepMV-CH2 genotype was present in all 101 samples, 23 of which were asymptomatic samples. Fifty-four samples had positive PepMV-CH2 diagnostics results before HTS, and 45 new identification cases were confirmed with RT-qPCR on the original plant sample material (Figure 4, Supplementary Table S1). The PepMV-EU and PepMV-LP genotypes were identified in 12 and 18 samples, respectively (Figure 4, Supplementary Table S1), in all cases in mixed infections with PepMV-CH2 (Figure 3, Supplementary Table S1). PepMV-EU was identified in 3 asymptomatic samples and PepMV-LP in 2. Supplementary Figure S1 presents the reads allocation for each PepMV genotype. The HTS study revealed 11 cases of mixed infections between PepMV-CH2 and PepMV-EU, previously unidentified by diagnostics, all of which were confirmed by RT-qPCR. Additionally, 13 new cases of PepMV-CH2 and PepMV-LP mixed infections were identified, 10 of which were confirmed with RT-qPCR (Figure 4, Supplementary Table S1). In 2 HTS identification cases of PepMV-CH2, and 3 of PepMV-LP, the presence of the virus could not be confirmed with a strain-specific RT-qPCR. ToBRFV was identified in 44 samples, of which 5 were asymptomatic (Figure 3). Positive diagnostics results were available prior to HTS for 31 of those cases (Figure 4, Supplementary Table S1). As previously mentioned, positive ToBRFV cases were preferentially selected for our study to facilitate the phylogenetic analysis of the virus. The proportion of positive ToBRFV samples identified is not directly indicative of the viral incidence in the countries of origin. The HTS study revealed 13 new cases for ToBRFV, 11 were confirmed with RT-qPCR and 2 samples showed discrepancies in the analysis.

Despite minimal prior detection, STV was also found in several of the analyzed samples (Figure 3 and Figure 4). The virus was found in 32 samples, 9 of which were asymptomatic (Figure 4, Supplementary Table S1). Twenty-seven of the newly identified cases were confirmed with RT-qPCR, while for 3 other samples, this virus had already been detected beforehand (Figure 4, Supplementary Table S1). Two HTS findings for STV could not be confirmed via the subsequent molecular assay (Figure 4). No discrepancies were identified for ToTV, with 2 cases detected in the total sample set (Figure 4, Supplementary Table S1). The nearly complete ToTV genome sequences were submitted to NCBI GenBank (PX434444 – PX434446). ToFBV was identified in 4 samples, 3 of which had positive diagnostics results before HTS (Figure 4, Supplementary Table S1). The presence of ToFBV in the fourth sample could not be confirmed with RT-qPCR. The nearly complete sequences for the 4 segments of ToFBV obtained from the 4 samples were aligned with publicly available sequences of the virus and the minimum nucleotide identity between all sequences was: 96.0% for RNA 1, 97.2% for RNA 2, 94.0% for RNA 3, and 95.4% for RNA 4. The nearly complete ToFBV genome sequences were submitted to NCBI GenBank (PX434430–PX434443). Lastly, CMV was found in 2 cases. The findings include 1 prior RT-PCR detection case and one case of discrepancy between HTS identification, and the following RT-qPCR performed on the original sample (Figure 4, Supplementary Table S1). The nearly complete CMV genome sequences were submitted to NCBI GenBank (PX434447–PX434449). Additionally to the 6 viral species of interest, a divergent tomato mild mottle virus strain (TMMoV, *Ipomovirus lycopersici*) was identified in two samples. The two sequences only showed 83.2% and 83.4% similarity to their most closely related relatives.

The tobacco vein clearing virus (TVCV, *Solendovirus venanicotianae*) is a member of the *Caulimoviridae* family and was detected in 22 samples (Figure 3). EVEs of members of the *Caulimoviridae* family have been identified as integrated into the genomes of many vascular plant species [49]. The TVCV assembled contigs for the 22 samples were aligned against the NCBI nt nucleotide sequence database using Blastn. The best alignment results for all 22 samples were tomato genomes, indicating the presence of a TVCV EVE in tomato.

### 3.2. Phylogenetic Analysis of Viral Species of Interest for Tomato Cultivation

**Figure 5:**
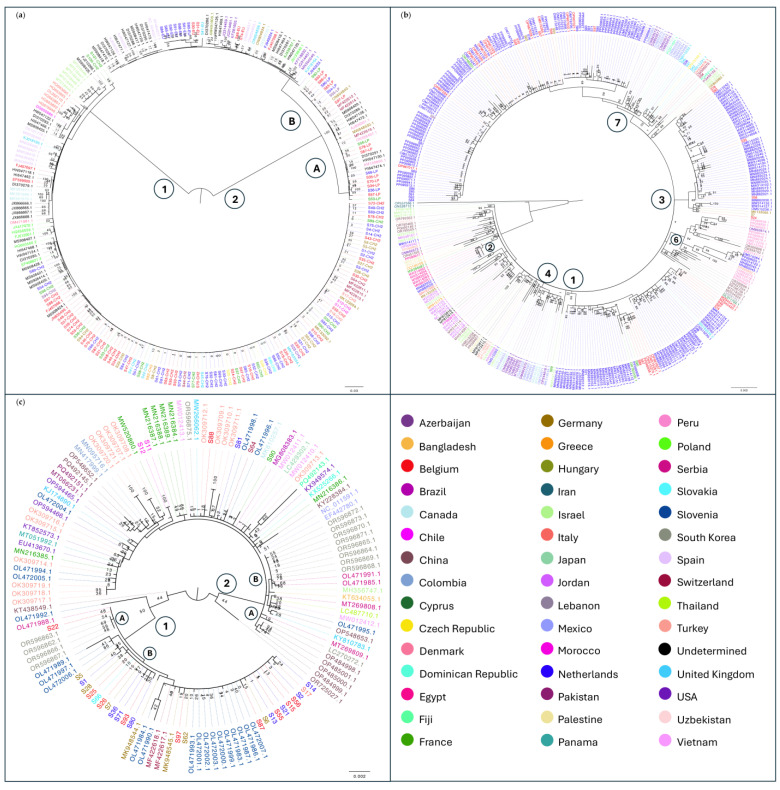
Phylogenetic analysis of viral species of interest for tomato cultivation. The publicly available sequences are labeled with the NCBI GenBank accession number. The HTS-obtained sequences are labeled with the sample number they were identified in. All sequences are colored depending on their country of origin. Each node is labeled with the obtained bootstrap values (**a**) PepMV maximum likelihood phylogenetic tree. The genotype name is added on the label of the HTS-obtained sequences. The two major clades are indicated by circled numbers next to the clades. The two subclades of Clade 2 are indicated with circled letters next to the subclades; (**b**) ToBRFV maximum likelihood phylogenetic tree. The six distinct clades identified in the present analysis in accordance with the ToBRFV Nextstrain build (v4) [51] are indicated with circled numbers next to the clades. The ToBRFV Nextstrain build numbering order is used. The nearly complete genome sequences S48, S67, S58, S57, S70, S63, S93, S62, S94 were published as OQ633211–OQ633213, OQ633215, OQ633216, OQ633218, OQ633220–OQ633222 in the ToBRFV Nextstrain build (v4) [51]. Sequences S87 and S86 were published as OR760198–OR760199 in Zisi et al., 2024 [52]; (**c**) STV maximum likelihood phylogenetic tree. The two major clades are indicated by circled numbers next to the clades. The subclades of clade 2 are indicated with circled letters next to the subclades.

The assembled contigs for all three genotypes of PepMV that had at least 70.0% coverage of the reference genomes were collected for all samples and the sequences were submitted to NCBI GenBank (PX316986–PX317053, PX317119). The HTS obtained nearly complete genome sequences were aligned with publicly available sequences representative of the three PepMV genotypes. This resulted in a PepMV collection of 246 sequences that shared at least 77.6% nucleotide identity among them. Within each genotype, sequences shared a higher percentage of identity, namely 96.2% for PepMV-CH2 sequences, 97.3% for PepMV-EU sequences, and 99.0% for PepMV-LP sequences. This PepMV collection was used to build a maximum-likelihood phylogenetic tree (Figure 5a). Two major clades were formed. Clade 1 included all the PepMV-CH2 sequences from the HTS study along with all the PepMV-CH2 public sequences of the collection (Figure 5a). Most of the PepMV-CH2 sequences obtained from the HTS study formed a single subclade (Figure 5a). Clade 2 of the phylogenetic tree had two subclades: 2A including all the PepMV-LP sequences, both public and identified in this study, and 2B containing all the PepMV-EU sequences in the PepMV collection of this study.

The contigs obtained for ToBRFV with genome coverage ≥70.0% were collected, and the sequences were submitted to NCBI GenBank (PX317054–PX317086, OQ633211–OQ633213, OQ633215, OQ633216, OQ633218, OQ633220–OQ633222, OR760198–OR760199). Due to its detrimental effects on tomato cultivation, ToBRFV has been studied extensively, with particular interest in its phylogeny. The Dutch National Plant Protection Organization (NPPO) created a build for tracking the ToBRFV outbreaks in the Nextstrain platform, where viral sequences could be submitted globally and facilitate a concise and comprehensive phylogenetic study of ToBRFV [50]. The ToBRFV HTS sequence collection was evaluated for redundancy to the already present database of the Nextstrain build. Nine nearly complete genome sequences (S48, S67, S58, S57, S70, S63, S93, S62, S94 corresponding to OQ633211–OQ633213, OQ633215, OQ633216, OQ633218, OQ633220–OQ633222) were already submitted in the latest version of the ToBRFV Nextstrain build (v4) [51]. Additionally, two more nearly complete genome ToBRFV sequences, obtained by this analysis, were published separately as part of a case of a ToBRFV mutation able to break the virus-specific resistance in new resistance tomato cultivars (S87, S86 corresponding to OR760198–OR760199) [52]. Furthermore, the HTS obtained nearly complete genome sequences aligned to publicly available ToBRFV sequences, building a ToBRFV collection of 372 sequences sharing at least 98.2% nucleotide identity. The collection was used to build a maximum-likelihood phylogenetic tree (Figure 5b). The results of the present phylogenetic analysis are in accordance with the ToBRFV Nextstrain v4 build. It was observed that ToBRFV sequences tended to form clades by geographical region, with 6 distinct clades identified (Figure 5b).

Contigs assembled for STV with reference genome coverage ≥70.0% were collected, and the sequences were submitted to NCBI GenBank (PX317087–PX317118). The HTS obtained nearly complete genome sequences were aligned with publicly available sequences, and an STV collection of 134 sequences was built and used to construct a maximum-likelihood phylogenetic tree. They shared 98.0% sequence identity. The tree had two major clades (Figure 5c). Clade 1 was divided into two subclades: Subclades 1A and 1B. Subclade 1A included 1 sample from the HTS study originating from BE, which clustered with sequences from South Korea (Figure 5c). Subclade 1B contained sequences from the HTS study collected from DE, NL, SK, CZ, BE, and IT. (Figure 5c). Similarly, Clade 2 is also divided into two subclades. Subclade 2A contains exclusively publicly available sequences. Subclade 2B includes both public and sequences identified in this study in samples from BE, NL, MA, CH, and FR (Figure 5c).

### 3.3. Viral Identification and Disease Etiology

**Figure 6:**
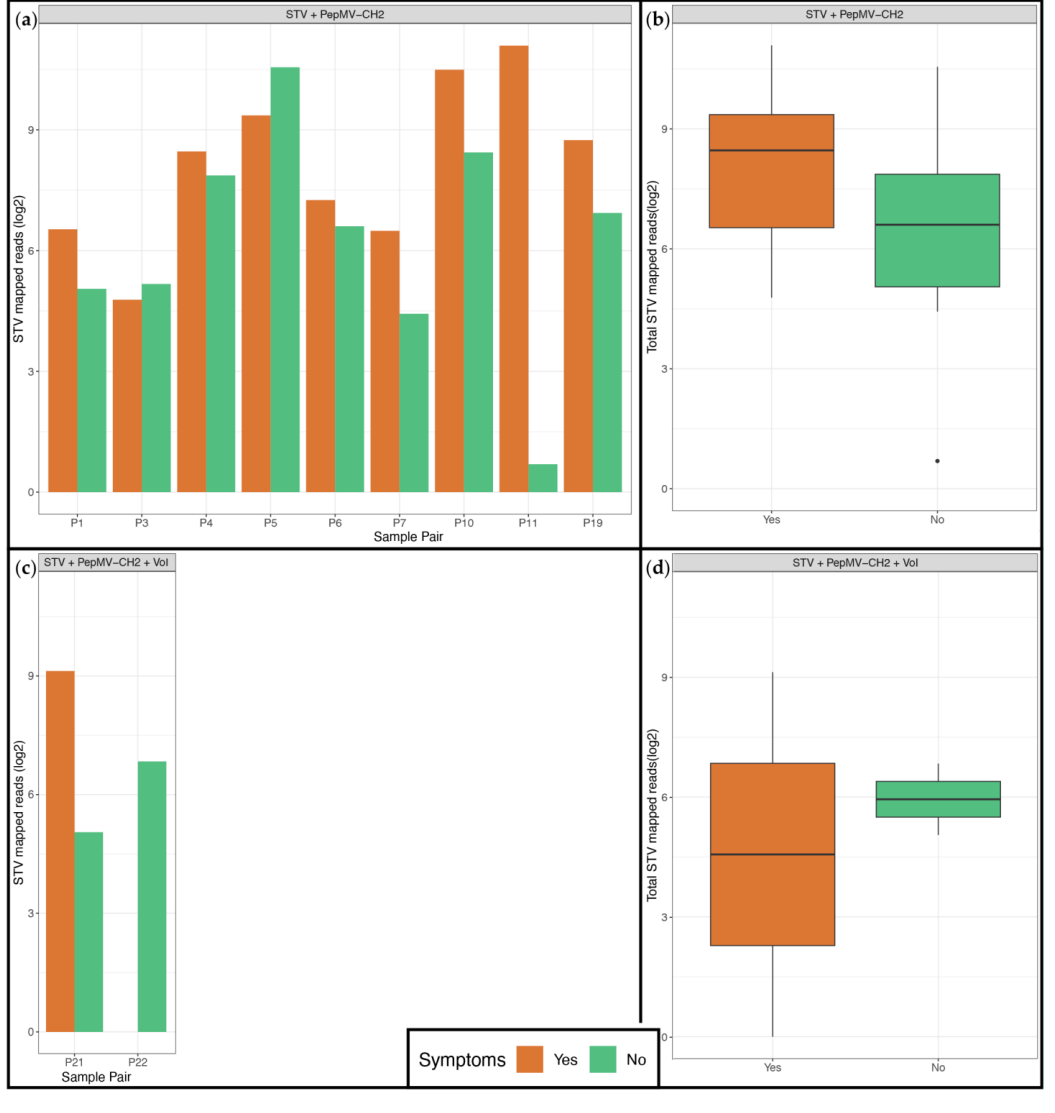
Association of STV presence with symptom presence. *x*-axis: Panels (**a**,**c**)—Pairs of symptomatic and non-symptomatic samples collected from the same commercial greenhouse, for which at least one member contained STV reads, Panels (**b**,**d**)—Total of symptomatic and non-symptomatic samples with STV identification; *y*-axis: Reads mapped on the STV genome on a logarithmic scale; the symptom status of samples is indicated in color: orange for presence and green for absence of symptoms. (**a**) Association of STV presence with symptom presence for samples with double infection (STV and PepMV-CH2) identification. Each of the 9 sample pairs is represented by two bars, one corresponding to the reads of the symptomatic sample (orange) and one corresponding to the reads of the non-symptomatic sample (green); (**b**) Association of STV presence with symptom presence for samples with double infection (STV and PepMV-CH2) identification. Boxes represent the total amount of STV reads obtained for each symptom status, the mean number of reads is indicated with a horizontal black line, Wilcoxon signed-rank test revealed no statistically significant difference between the two mean values (*p*-value: 0.1); (**c**) Association of STV presence with symptom presence for samples with identification of triple infection (STV, PepMV-CH2 and one additional virus of interest. Each of the 2 sample pairs is represented by two bars, one corresponding to the reads of the symptomatic sample (orange) and one corresponding to the reads of the non-symptomatic sample (green); (**d**) Association of STV presence with symptom presence for samples with identification of triple infection (STV, PepMV-CH2 and one additional virus of interest). Boxes represent the total amount of STV reads obtained for each symptom status, and the mean number of reads is indicated with a horizontal black line, The Wilcoxon signed-rank test revealed no statistically significant difference between the two mean values (*p*-value: 0.06).

The sample collection included 24 pairs of symptomatic and non-symptomatic samples collected from the same commercial greenhouse. Eleven sample pairs of the same type (leaf, fruit, sepal) had STV found in at least 1 member of the pair. In all pairs, STV was co-identified with PepMV-CH2, and in 2 pairs an additional virus of interest was identified. The association between the presence of STV in a plant and symptom presence was examined by comparing the obtained amount of reads from symptomatic and non-symptomatic samples, both per pair and overall, taking the co-infecting viruses into consideration (Figure 6). No clear trend was observed towards STV presence or increased read identification and the presence or absence of symptoms, neither in cases with 2 viruses (Figure 6a) nor in cases with 3 viruses present (Figure 6b). Comparison of the total STV reads obtained from symptomatic and non-symptomatic samples did not result in statistically significant differences both for 2 and 3 virus identification cases (*p*-value: 0.1 and 0.06 respectively) (Figure 6).

**Figure 7:**
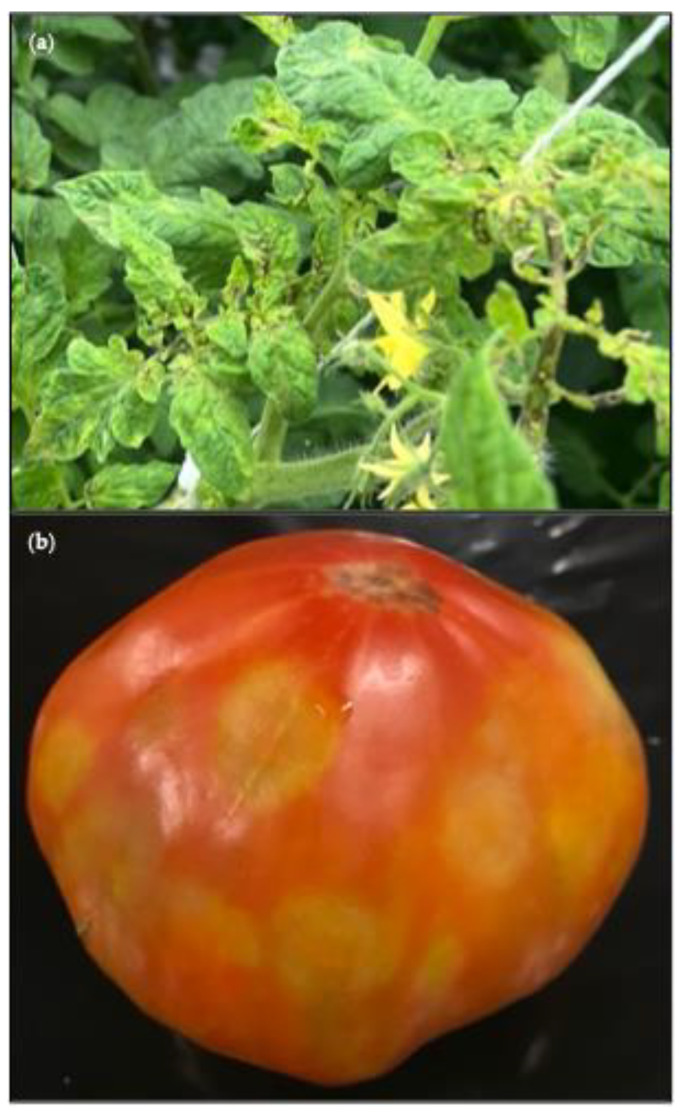
(**a**) ToTV leaf symptoms. The picture presents plants presenting a characteristic symptom of ToTV infection: leaf necrosis (dark spots) on the head (top part) of the plant, light deformation (slight curling or cupping of the leaves), blistering, and mosaic discoloration on the leaves. Leaf sample S99 was collected from the presented plants; (**b**) ToFBV fruit symptoms. Sample S98 was collected from the presented fruit. The picture presents the blotchy ripening (uneven ripening circular spots) observed in fruits with ToFBV infection.

ToTV was identified in 2 leaf samples, S43 and S99, collected in 2020 and in 2024 respectively. The plants that were sampled both had necrotic spots in the leaves in the head of the plant, light leaf deformation (slight curling or cupping of the leaves), as well as blistering and mosaic discoloration on the leaves (Figure 7a). They also both had open fruits. The symptoms directed the diagnostics analysis to frequently occurring economically important viruses that typically induce these symptoms such as tobamoviruses or TSWV. However, the molecular assays were negative for these viruses. Hence the samples were included in the HTS analysis in an effort to clarify the symptom cause. ToTV is indeed also known to cause necrotic spots, leaf deformation, and open fruits in tomato plants [53], and its presence was confirmed with an RT-qPCR on the original sample material.

ToFBV was identified in 4 fruit samples: S78 was collected in 2021, S96 was collected in 2023, and S98 and S100 in 2024. The symptoms of S78 were similar to ToBRFV-induced symptoms, hence the requested diagnostics analysis was for ToBRFV. When the presence of ToBRFV was confirmed in the sample, it was not further tested for ToFBV. HTS however revealed the presence of both ToBRFV and ToFBV in the sample. Samples S96, S98, and S100 showed uneven blotchy ripening with circular spots (Figure 7b) and tested positive for ToFBV with RT-qPCR before HTS.

TMMoV was identified in 2 fruit samples, S9 and S10, collected in the same commercial greenhouse in 2018. S9 had marbling discoloration while S10 was an asymptomatic sample collected as a ‘healthy’ control for S9. PepMV-CH2 and STV were also identified via HTS in both samples.

## 4. Discussion

Tomato cultivation has had a steady rise in both the dedicated area and production yield over the last decade [6]. Although commercial greenhouses provide a degree of control in the cultivation environment, plant viruses can still have a big impact on production yield and can lead to reductions of 42.1% up to 95.5% [54]. Due to the significant consequences viral outbreaks can have, this study aimed to explore the virome composition of commercial tomatoes using viral metagenomics approaches. The samples included in the study were selected either because of conclusive diagnostics results of epidemiological interest (e.g., ToBRFV infection cases) or due to the presence of symptoms combined with inconclusive diagnostics. For the latter category, non-symptomatic samples, collected from the same greenhouse, were included as a ‘healthy’ control if available. The selection process led to a set of 101 samples, collected from 13 countries, over a period of 7 years. The sample set was processed using the VLP enrichment protocol NetoVIR [25] and deep sequenced. The results were analyzed with the ViPER pipeline [26]. It should be noted that the viral identification percentages of this study are highly affected by the sample selection rationale and are not directly representative of the viral prevalence in the included countries.

In total 43 viral species were identified, belonging to 25 families, the majority of which had plants, fungi, and insects as most likely hosts. Six viral species of significant interest to the tomato value chain were found: PepMV, ToBRFV, STV, ToTV, ToFBV, and CMV. Interestingly, we did not find any novel pathogenic virus species and the median of viral species identified per sample was 2. Many commercial greenhouses are applying cross-protection strategies against natural infections with aggressive PepMV isolates, using mild PepMV isolates [10]. It has been shown that the use of NetoVIR results in a good correlation between the obtained HTS reads and viral load [25], leading in some cases of highly infectious and persistent viruses, such as PepMV and ToBRFV [7,55], to their dominance in the resulting sequence reads, thus potentially masking the presence of viruses present in lower titers [10].

Despite the low viral identification median, for one sepal sample (S16) 26 viral species were identified. These species belonged to 18 viral families, of which 10 had fungi as hosts, 4 had fungi and plants, and 1 had fungi and insects. These results could indicate the presence of a fungal infection in the sampled plant. 

The identification of the 6 viral species of interest for tomato cultivation (PepMV, ToBRFV, STV, ToTV, ToFBV, CMV) was coupled with RT-qPCR results, either from pre-existing diagnostics results or performed after HTS. For 10.9% of the cases, the results of HTS and RT-qPCR were not in accordance (Supplementary Table S1). HTS findings with low viral relative abundance often corresponded to RT-qPCR results close to the assay’s detection limit. Additionally, sample type and the location of sampling on the plant played a role in some of these cases. Different leaves from the same infected plant may contain distinct levels of virus. For example, ToBRFV-infected plants have been found to have higher viral concentrations in younger leaves in the head of the plant than in older leaves [56]. In cases where mixed leaf samples were collected, this can result in differences in the testing, especially in the case of viral titers close to the detection limit of the different assays used.

An additional advantage of viral metagenomics is that the obtained sequences can be used to study the phylogeny of viruses of interest [50]. In the present study, this was applied to PepMV and ToBRFV, two viruses with worldwide distribution [13,57], that were the most prevalently identified viral species in this study. For PepMV three genotypes were identified, PepMV-CH2 in 100.0%, PepMV-EU in 11.9% and PepMV-LP in 17.8% of this predominantly European sample set. PepMV-CH2 is the most dominant genotype in Europe [10]. Additionally, mild isolates of all three genotypes are used in cross-protection strategies against PepMV in commercial tomato cultivation [58]. A previous study in Belgium found the occurrence of PepMV-EU and PepMV-LP at 7.0% and 10.0% respectively [10]. In our study, PeMV-CH2 was the sole PepMV genotype identified in 71 samples, while PepMV-EU and PepMV-LP were always identified in mixed infections with PepMV-CH2 in 18 and 12 samples respectively. Two studies, in Spain and in Belgium, have shown recombination events between PepMV-CH2 and PepMV-EU in some mixed infection cases [34,59]. The phylogenetic analysis for PepMV revealed the formation of two major clades, one consisting of the PepMV-CH2 sequences and one splitting into two subclades that consist of the PepMV-EU and PepMV-LP sequences respectively. None of the sequences identified in this study fell between the main clades, suggesting that recombination most likely did not occur in our sample set. All 246 PepMV sequences shared 77.6% nucleotide sequence identity among them, a percentage that is in accordance with previous studies showing that PepMV-CH2 shares 78.0%—80.0% sequence identity with PepMV-EU and PepMV-LP [10]. ToBRFV was identified in 43.6% of the sample set. It is important to note that the identification rate of ToBRFV in this study is highly affected by the selection of positive cases to facilitate the phylogenetic analysis of the virus and is not representative of the viral incidence in the included countries. The diversity between ToBRFV sequences is low, with the 372 analyzed sequences sharing at least 98.2% nucleotide identity. The phylogenetic analysis of the acquired sequences combined with publicly available sequences resulted in similar observations as the ToBRFV Nextstrain build (v4) created by the Dutch NPPO [51]. An earlier Nextstrain build had identified 8 ToBRFV clades [60], whereas in the latest update (v4) a large number of added sequences led to changes in clade 5 which collapsed due to insufficient support. This clade does not appear as a separate clade in the updated phylogenetic tree anymore. In addition, clades 6 and 8 were merged to form clade 6 [51]. Thus, the six clades currently recognized in the tree are Clades 1, 2, 3, 4, 6, and 7 [51].

The HTS analysis also revealed the presence of STV. The virus belongs to the genus of *Amalgavirus* and the family of *Amalgaviridae* [61]. Although the first report of STV dates back to 2009 [62], its current distribution is not well-characterized, since it can cause both symptomatic and asymptomatic infections in tomato plants [61]. STV has a high rate of vertical transmission through seeds, while there is no evidence for horizontal transmission between plants [61]. It was identified in 32 of the analyzed samples (31.7%). Diagnostics results were available only for 3 of these samples, indicating that STV can frequently be present in a commercial greenhouse without clear signs of infection. The sample selection rationale for this study in combination with the limited reporting on the virus, due to asymptomatic infections, makes it difficult to assess whether this rate is representative of the viral incidence in the included countries. A study conducted in Turkey that included both symptomatic and non-symptomatic samples, showed an STV prevalence of 54.3% [61]. The STV sequences obtained in our study were combined with publicly available sequences to study their phylogeny. The 134 sequences had a minimum identity of 98.0%. The maximum-likelihood phylogenetic tree showed two major clades, Clade 1 and Clade 2 which correspond to the two major clades (II and I respectively) identified in the study conducted in Turkey [61]. These results showcase that HTS can be a powerful tool for the identification of viruses that can circulate undetected due to their (lack of) symptomatology. It can also provide some insights into the diversity and phylogenetic relationships of understudied viruses.

Additionally, STV is frequently identified in mixed infections, and some studies have shown that the virus can aggravate the symptoms of the co-infecting virus [29,61]. This, in combination with the observation that the virus is frequently present in a single infection without any symptoms, compelled the study of the association between symptom status and the presence of STV. As shown in Figure 6, our analysis did not show an association between symptom status and the abundance of STV in the samples, although it must be acknowledged that our sample numbers were limited. The role of STV in symptom development requires further examination.

ToTV was identified in 2 symptomatic leaf samples collected in 2020 and 2024, in 2 different greenhouses. ToTV is a member of the *Torradovirus* genus of the *Secoviridae* family [53]. It has been shown to be transmitted via whitefly species [63]. Tomato is the natural host for ToTV, while several weeds have also been identified to act as alternative hosts for the virus [63]. In Europe, the first reports of the virus were in 2007 in Poland [64], in 2009 in France [65] and Hungary [66], and in 2010 in Italy [67] and Spain [68]. As the majority of commercial tomato cultivars include resistance genes for ToTV, outbreaks of this virus are no longer abundantly present in Europe’s tomato cultivation [63,69,70]. The 2 leaf samples exhibited the typical ToTV symptoms in tomatoes, which are severe necrotic spots on the leaf base and on fruits as well as leaf deformation [53]. However, these symptoms can also be observed in cases of infection with other more frequently occurring tomato viruses such as ToBRFV [71] or TSWV [19], which were the initially performed diagnostics tests on the 2 symptomatic samples in this study. After the diagnostics tests were negative for the more frequent viruses, HTS was performed which allowed the identification of ToTV as the causal agent for the symptoms. The tomato plant that was sampled in 2024 was a cultivar that did not carry the common resistance against ToTV. No cultivar details are available for the other plant sample but likely also in this case no resistance was present in the cultivar. As the samples were identified four years apart and no other tomato samples with similar symptoms were analyzed in our laboratory neither in 2020 nor in 2024, these new ToTV identification cases appear to be isolated. In a study in South Africa, it was observed that weeds such as *Datura stramonium* and *Abutilon grantii* Meeuse can act as a reservoir of ToTV which in areas that combined high vector population and susceptible cultivars led to infection cases in tomatoes [72]. As weeds of the *Datura* genus and *Datura stramonium* itself are present in Europe, it is possible that these two samples represent similar cases where ToTV was present in a reservoir host and eventually infected the cultivars without resistance. Sampling of weeds in areas surrounding commercial greenhouses could give a clearer view of the ToTV incidence in Europe.

The HTS analysis also led to the identification of another whitefly-transmitted virus, TMMoV. TMMoV belongs to the genus *Ipomovirus* of the family *Potyviridae* and has mild effects on tomato plants mainly causing mottling symptoms on leaves [73,74]. In the present study, the virus was identified in a symptomatic and a non-symptomatic sample collected in 2018. ToTV and TMMoV are the only whitefly-transmitted viruses identified in this virome analysis, most likely because the majority of analyzed samples were collected from commercial greenhouses in Western Europe. Although whitefly-transmitted viruses constitute a significant threat to tomato cultivation on a worldwide scale, vector control becomes more difficult in warmer and drier climates [5,72].

ToFBV belongs to the *Blunervirus* genus of the *Kitaviridae* family and has been identified in Europe, Brazil, and Australia [75]. In most cases, the identification of ToFBV coincided with the identification of tomato russet mite (*Aculops lycopersici* Massee) infestations, which most likely function as the vector for the virus [75,76,77]. Tomato russet mite is considered a pest for *Solanaceae* crops and has been identified in both tropical and temperate climates globally [75,78,79]. The viral symptoms are very characteristic, with fruits showing uneven, blotchy ripening and dimpling of fruits (Figure 7b) [75], which steered the diagnostics analysis of samples in this study showing such symptoms. It allowed the identification of the virus before HTS for 3 out of the 4 samples where ToFBV was found. Comparison of the obtained ToFBV sequences for each segment with publicly available sequences revealed high identity amongst the sequences. The sample without prior detection of ToFBV, showed symptoms indicative of a ToBRFV infection which was confirmed with diagnostics, and analysis was completed. This sample showcases how HTS analysis can provide unique and useful insights into the tomato virome. Through HTS the presence of additional viruses can be identified, in cases where routine analysis would be concluded after detection of a virus that is likely causing the observed symptoms. In the present study in general, very few insect-transmitted viruses were identified in a small number of cases. This is most likely linked to the fact that the majority of analyzed samples were collected from active greenhouses which are quite closed off from the environment and where effective insect pest management is more feasible.

The HTS analysis also led to the identification of CMV. CMV belongs to the *Cucumovirus* genus of the family *Bromoviridae* [80]. The virus is transmitted by aphid species and has a worldwide distribution [80,81]. CMV can cause severe yield losses in tomato cultivation and its symptoms include mosaic, chlorosis, and leaf malformation [80,82]. The virus was identified in 2 symptomatic leaf samples collected in 2020 and 2022.

EVEs are virus-derived sequences that are integrated into the viral host genomes [49]. In plants most characterized EVEs concern members of the family *Caulimoviridae* [49]. Caulimoviruses are reverse-transcribing viruses or virus-like retrotransposons with double-stranded DNA genomes [83]. TVCV belongs to the *Caulimoviridae* family, and an endogenous form of the virus has been identified in the tomato genome and is also recognized to have pathogenic potential [84,85]. A host read subtraction step in the bioinformatics pipeline could provide a useful differentiation between the detection of an EVE and a viral infection finding. In the present study, host reads were not removed leading to the assembly of TVCV contigs for 22.8% of the sample set. All contigs were aligned with the NCBI nt nucleotide database using Blastn resulting in tomato genomes as best alignments, indicating that the findings were EVE derived.

HTS is a powerful tool that in recent years HTS has been increasingly used for viral discovery and detection, and to study virus diversity in tomatoes [1]. A major challenge in viral metagenomics studies is the selection of a sampling processing protocol and tools for the bioinformatics analysis, as these steps determine the resolution and focus of obtained results and should be tailored to the posed research questions despite the absence of methodological standards [1,21]. In the present study, the VLP enrichment protocol NetoVIR [25] was selected which resulted in a higher ratio of viral to plant reads for most of the analyzed samples. Viral metagenomics analysis also allows the monitoring and phylogenetic study of both well-established and less common viruses that might spread undetected by causing asymptomatic infections. Additionally, HTS can be a useful diagnostics tool for rarely occurring viruses, mixed infections, or cases where symptomatology is not clearly indicative of a specific virus. Generating comprehensive guidelines for the selection of processing and reporting protocols can improve the variation between studies and the efficiency of results interpretation.

## Data Availability

The raw sequencing data were submitted to the NCBI Sequence Read Archive (SRA) with a BioProject accession number: PRJNA1329211. Complete viral genome sequences were submitted to the NCBI GenBank database under accession numbers: PX316986–PX317119, PX434430–PX434449. Only unique PepMV-CH2 sequences were submitted to the NCBI GenBank database. One representative complete genome (in bold) was submitted for the following three groups of identical PepMV-CH2 sequences: (i) **S1**, S2, S4, S5, S6, S9, S10, S11, S12, S13, S14, S15, S18, S22, S25, S28, S33, S35, S36, S37, S52, S60, S68, S69, S71, S73, S75, S78, S81, S89, S90, S92, (ii) **S8**, S42, S44, S65, (iii) **S16**, S20, S26, S29, S31, S32, S39, S40, S45, S47, S53, S56, S57, S58, S61, S62, S63, S66, S67, S70, S74, S76, S84, S86, S91, S93, S94, S98. The ViPER pipeline script can be accessed at https://github.com/Matthijnssenslab/ViPER, accessed on 25 November 2018. All the required code to reproduce the virome analysis will be made available at: https://github.com/Matthijnssenslab/Tomato_virome, accessed on 25 November 2018.

## Supplementary Materials

The following supporting information can be downloaded at: https://www.mdpi.com/article/10.3390/v17101334/s1, Table S1: Sample set overview with collected metadata and molecular detection for the viruses of epidemiological interest for tomato cultivation; Table S2: Molecular detection assays for the viruses of epidemiological interest for tomato cultivation; Table S3: Bioinformatics analysis overview and assembly details for phytopathogenic viral species; Table S4: Publicly available sequences used in phylogenetic analysis of PepMV, ToBRFV, and STV, and in diversity analysis of ToFBV; Figure S1: PepMV reads allocation in the three identified genotypes. *x*-axis: Analyzed samples; *y*-axis: Identified PepMV genotypes.
