# Supplementary figures and images for: Metagenomics Study of the Commercial Tomato Virome Focused on Virus Species of Epidemiological Interest
Source: Viruses. 2025 Sep 30;17(10):1334. doi: 10.3390/v17101334 (PMC12568099; doi:10.3390/v17101334)

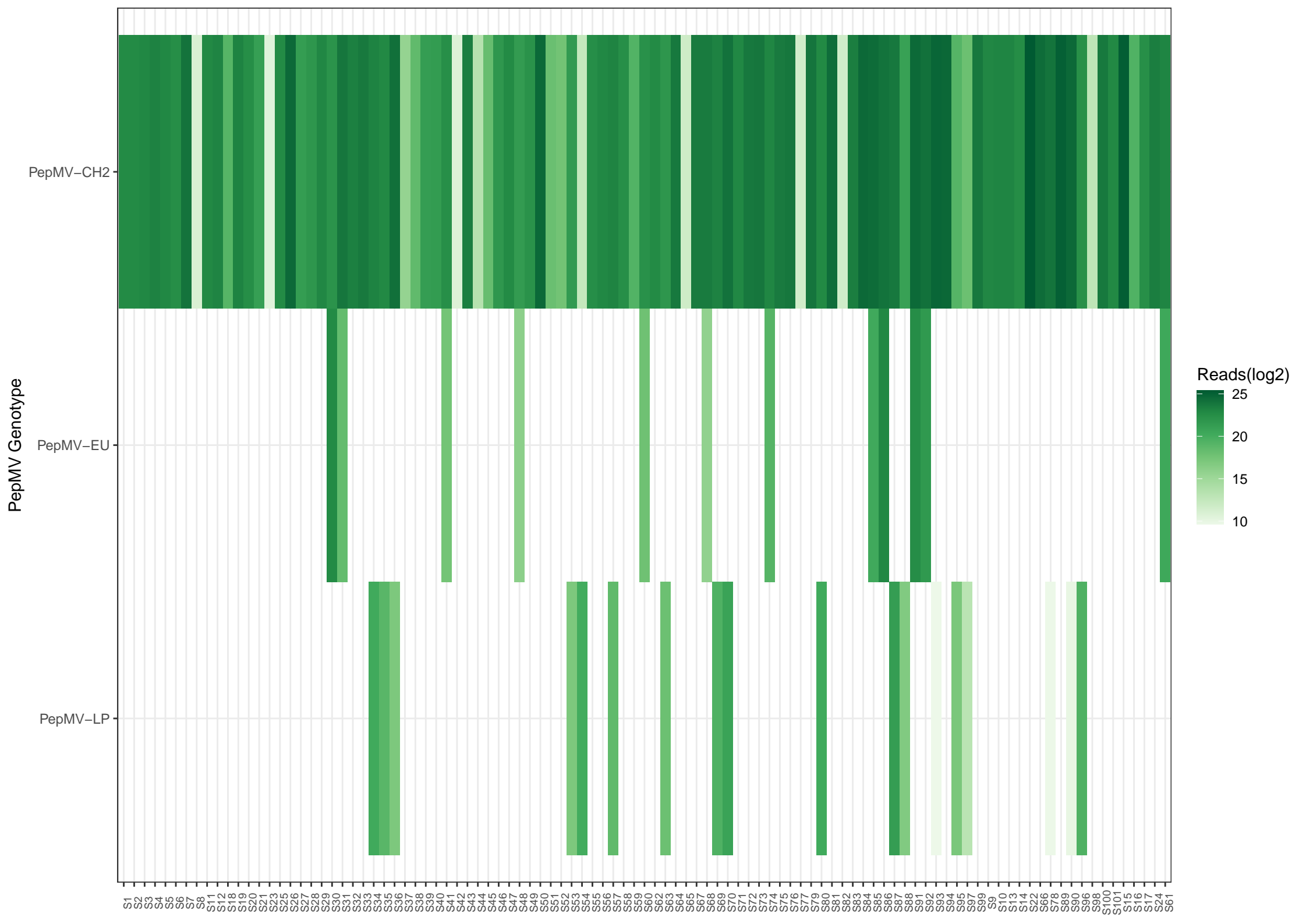

Supplement: Supplementary file 1 [file viruses-17-01334-s001.zip › Supplementary Figure S1.pdf]
